# Comparative Genomic and Biological Investigation of NADC30- and NADC34-Like PRRSV Strains Isolated in South Korea

**DOI:** 10.1155/tbed/9015349

**Published:** 2025-01-22

**Authors:** Haemin Jeong, Youngjoon Eo, Duri Lee, Guehwan Jang, Kyeng-Cheol Min, An Kook Choi, Hokeun Won, Jungjoon Cho, Sang Chul Kang, Changhee Lee

**Affiliations:** ^1^College of Veterinary Medicine and Virus Vaccine Research Center, Gyeongsang National University, Jinju 52828, Republic of Korea; ^2^Nawoo Veterinary Group, Yangsan 50573, Republic of Korea; ^3^ChoongAng Vaccine Laboratories, Daejeon 34055, Republic of Korea; ^4^SoJung Animal Hospital, Yesan 32416, Republic of Korea; ^5^Optipham Inc., Cheongju 28158, Republic of Korea

## Abstract

Porcine reproductive and respiratory syndrome virus (PRRSV) is a globally endemic, costly swine arterivirus with wide genetic and antigenic variations, leading to the frequent appearance of novel virulent strains that hampers PRRSV control. Recently, NADC30-like (lineage 1C, L1C) and NADC34-like (lineage 1A, L1A) PRRSV strains were reported to be prevalent in mainland South Korea and became the main epidemic strains persistently attributed to PRRSV outbreaks nationwide, raising great concern in the domestic pork industry. Although the genotypic and pathotypic variability of NADC30- and NADC34-like viruses has been explored in the United States and China, their genomic and biological characteristics have been scarcely studied in South Korea. Here, NADC34-like GNU-2353 and NADC30-like GNU-2377 strains were independently identified from vaccinated swine herds experiencing high piglet mortality. Whole-genome sequencing and phylogenetic analysis revealed that GNU-2353 and GNU-2377 clustered into sublineages L1A (NADC34-like) and L1C (NADC30-like), respectively, sharing high genomic homology with their corresponding lineage-representative strains and harboring the same molecular signatures of continuous 100 and discontinuous 131 amino acid deletions in the nsp2-coding region, respectively. Recombination detection indicated that GNU-2353 and GNU-2377 were recombinants and evolved through natural interlineage recombination between NADC34-like (L1A, major parent) or NADC30-like (L1C, major parent) and RespPRRS modified live virus (MLV)–like (lineage 5, minor parent) strains, respectively. Both viruses displayed homogenous growth kinetics but replicated faster than the prototype VR-2332 in a porcine alveolar macrophage cell line (PAM-KNU). The transcriptional profiles of immune response genes in infected PAM-KNU cells varied between the isolates and VR-2332; particularly, interleukin-10 expression was dramatically upregulated in cells infected with GNU-2353 and GNU-2377. Piglets with GNU-2353 and GNU-2377 infection had high fever; weight loss; increased viremia and nasal shedding; viral distribution in various tissues; thymic atrophy; and apparent macroscopic and microscopic lung lesions, including interstitial pneumonia and viral colonization, compared with control piglets, suggesting that both isolates were virulent to pigs. Remarkably, GNU-2353 caused higher fever, mortality rate (40%) with cyanosis, viremia, and viral shedding within 2 weeks and significantly higher viral loads in several organs than GNU-2377 infection. Thus, NADC34-like GNU-2353 was more pathogenic than NADC30-like GNU-2377. Our findings provide insights into the current epizootic circumstance of NADC30- and NADC34-like PRRSV in South Korea and can aid in tailoring improved control strategies.

## 1. Introduction

Porcine reproductive and respiratory syndrome (PRRS), characterized by reproductive disorder in breeding animals and respiratory illnesses in pigs of all ages, is a financially significant viral disease that has posed an ongoing threat to global pig production [1, 2]. PRRS virus (PRRSV), the causative pathogen of PRRS, is an enveloped, single-stranded, positive-sense RNA virus that belongs to the genus *Betaarterivirus*, family *Arteriviridae*, and order *Nidovirales* [3, 4]. PRRSV possesses a polycistronic genome of ~15 kb containing a 5′-untranslated region (UTR), 11 open reading frames (ORFs; ORF1a, ORF1b, ORF2a, ORF2b, ORFs 3–7 with ORF5a, and a short transframe ORF in the nsp2 region), and a 3′-UTR. ORF1a and ORF1b encode two replicase polyproteins that are posttranslationally cleaved into at least 16 nonstructural proteins (nsp1*α*, nsp1*β*, nsp2, nsp2TF, nsp2N, nsp3–nsp6, nsp7*α*, nsp7*β*, and nsp8–nsp12). The downstream ORFs encode eight structural proteins, including five envelope-associated glycoproteins (GP2–GP4, GP5a, and GP5), two nonglycosylated membrane proteins (E and M), and one nucleoprotein (N) [5–7].

Given its great genetic and antigenic heterogeneity, PRRSV was classified into two distinct species, which were renamed as *Betaarterivirus europensis* (PRRSV-1) and *Betaarterivirus americense* (PRRSV-2) by the International Committee on Taxonomy of Viruses in 2023 (MSL39.v3), according to the geographic locations where the viruses (represented by the Lelystad and VR-2332 strains) were first isolated [3, 8]. Based on ORF5 sequence, PRRSV can be further divided into four subtypes within PRRSV-1 and 11 monophyletic lineages (L1–L11) and 21 sublineages (sublineages 1A–1F and 1H–1J in L1, sublineages 5A and 5B in L5, sublineages 8A–8E in L8, and sublineages 9A–9E in L9) within PRRSV-2 [9, 10].

In South Korea, both species have cocirculated in swine herds since PRRSV-1 was identified in 2005 [11, 12]. However, PRRSV-2 has been popular across the nation and has continued to accumulate its genetic diversity through independent evolution for several decades, thereby forming nation-specific clades, namely lineages KOR A (LKA, recently reclassified as L11), B (LKB), and C (LKC corresponding a South Korean [KOR] branch of L1 and recently reclassified as L1J) [10, 13, 14]. Thus, most PRRSV-2 strains in South Korean pig populations belong to L1 (MN184-like), L5 (VR-2332-like), L8 (Fostera vaccine-like), and three KOR lineages [13–15]. L1 and vaccine-variant L5 represent the most prevalent PRRSV-2 strains circulating in the domestic pig farms [13, 15, 16]. In particular, L1 became the largest population of PRRSV-2 strains detected in South Korea, and its virulent derivatives with novel genotypic and augmented pathotypic traits have continued to emerge, causing substantial damage to the swine [15, 17].

The first case of virulent L1 PRRSV-2 appeared in Canada in the 1990s, and the strain then spread through the United States, where it has become the most dominant and divergent lineage, with nine sublineages (1A–1F and 1H–1J) [10, 18, 19]. In the early 2000s, an outbreak of L1 PRRSV-2 began in North America with sublineage L1F (formerly L1.9), representative strain MN184 [20]. In 2013, sublineage L1C (formerly L1.8, representative strain NADC30) emerged in Canada and sequentially spread in the United States and China in the same year [21, 22]. Subsequently, large-scale epidemics of sublineage L1A (formerly L1.5, representative strain NADC34), characterized by abortion storms in sows and high mortality in piglets, occurred in the United States in 2014 [23, 24], in Peru in 2015 [25], and in China in 2017 [26]. In South Korea, the occurrence of NADC30-like (L1C) and NADC34-like (L1A) PRRSVs was first reported in 2014 and 2022, respectively [16, 27]. Afterward, sublineages L1A and L1C became widespread and caused the recent PRRSV epidemics in mainland South Korea, excluding Jeju Island, with high clinical and economic impact on domestic pig herds. In this study, we independently identified NADC30-like (L1C) and NADC34-like (L1A) PRRSV strains from pig farms with high PRRS-associated mortality in piglets. The present study aimed to determine the complete genome sequences of these two strains and characterize their genotypic traits. We also isolated NADC30- and NADC34-like viruses, GNU-2377/KOR/2023 and GNU-2353/KOR/2023, circulating in South Korea and compared their phenotypes in vitro and in vivo.

## 2. Materials and Methods

### 2.1. Clinical Cases and Sample Collection

In late 2023, suspicious PRRS cases accompanied by high mortality (loss of maximum 30% weaned and growing piglets) were reported independently in a 900-sow farrow-to-finish farm (farm A) in Chungnam Province and a 16,000-pig wean-to-finish farm (farm B) in Jeonbuk Province, with Ingelvac PRRS modified-live vaccine (Ingelvac modified live virus [MLV])-immunized animals (Boehringer Ingelheim Vetmedica, St. Joseph, MO). Clinical lung (farm A) and serum (farm B) samples were collected from dead and viremic 60-day-old pigs suspected of PRRSV infection, respectively, and submitted to our laboratory for diagnosis. Lung samples were homogenized into 10% (wt/vol) suspensions with phosphate-buffered saline using MagNA Lyser Instrument (Roche Diagnostics, Mannheim, Germany) in three 15-s rounds at 8000 × *g*, and the suspensions were centrifuged for 10 min at 4500 × *g* (Hanil Centrifuge FLETA5, Incheon, South Korea), as described [28]. The clinical samples were first tested with real-time RT-PCR (rRT-PCR) using One Step TB Green PrimeScript RT-PCR Kit (TaKaRa, Otsu, Japan), as described in previous research [17]. The reaction mixtures were amplified using CronoSTAR 96 Real-Time System (Clontech, Mountain View, CA), and the results were analyzed using software, as outlined elsewhere [29]. rRT-PCR-positive RNA samples with the lowest cycle threshold (Ct) value were further subjected to RT-PCR for ORF5 amplification and nucleotide sequencing, as described previously [30]. PRRSV-positive clinical specimens were filtered through a 0.22-μm-pore syringe filter (Millipore, Billerica, MA) and stored at −80°C until genomic sequencing and virus isolation.

### 2.2. Whole-Genome Sequencing (WGS) and Phylogenetic Analysis

The full-length genomic sequences of NADC30- and NADC34-like PRRSV-2 isolates were determined using the Sanger method, as described previously [17]. Eight overlapping cDNA fragments covering the entire viral genome were amplified using RT-PCR from each clinical sample, labeled original “passage 0 (P0)” stock, and sequenced, as outlined in previous protocols [17, 31]. The 5′-and 3′-terminal sequences of the viral genome were determined using rapid amplification of cDNA ends methodology, as described [32]. The complete genomic sequences of NADC34- and NADC30-like strains, designated GNU-2353-P0 (L1A) and GNU-2377-P0 (L1C), were deposited in GenBank under accession numbers PQ201859 and PQ201860, respectively. The sequences of full genomes, nsp2, and ORF5 obtained in this study, in addition to sequences of lineage-representative PRRSV-2 strains retrieved from the GenBank database, were used for multiple sequence alignments and phylogenetic analyses, as outlined previously [17, 33, 34].

### 2.3. Recombination Analysis

Potential recombination events were analyzed using recombination detection program (RDP), SimPlot, and phylogeny, as described previously [17]. In brief, eight algorithms (RDP, GENECONV, BootScan, MaxChi, Chimaera, SiScan, 3Seq, and LARD) from RDP4 software (version 4.101) were first applied to detect recombination signals and hot breakpoints [35]. Recombination events were considered present when detected by at least five of the aforementioned algorithms. Next, Simplot software (version 3.5.1) was selected to verify recombination signals and breakpoints. Phylogenetic analysis based on recombination regions was performed to confirm putative recombination data.

### 2.4. Virus Isolation

PRRSV was isolated from PRRSV-positive samples using immortalized porcine alveolar macrophage (PAM) cells (PAM-KNU; [36]), as described [17]. Briefly, PAM-KNU cells were inoculated with each prepared inoculum and monitored daily for cytopathic effects (CPE). When ~70% of the inoculated cells exhibited CPE, viruses were harvested and freeze–thawed three times. The clarified supernatants were aliquoted and stored at −80°C as “passage 1 (P1)” viral stock (GNU-2353-P1 and GNU-2377-P1) for use in WGS and subsequent serial passages. The passage 2 (P2) virus stock was plaque-purified in PAM-KNU cells three times to separate viral progeny derived from a single infectious virion. The P5 stock of each plaque-purified PRRSV strain (GNU-2353-P5 and GNU-2377-P5) was used for the experiments unless otherwise indicated. PRRSV isolation was verified by an immunofluorescence assay (IFA) using a monoclonal antibody (MAb) against PRRSV-2 N protein (CAVAC, Daejeon, South Korea), as outlined in previous protocols [17]. The growth curve of each strain was measured by virus titration, as described previously [36]. PAM-KNU cells were infected with PRRSV at a multiplicity of infection (MOI) of 1.0, as described above. Virus supernatants were harvested at 6, 12, 24, 36, and 48 h postinfection (hpi). Virus titers were quantified in triplicate on PAM-KNU cells using IFA and expressed as 50% tissue culture infectious dose (TCID_50_) using the Reed–Muench method [36]. The complete genomic sequences of P1 and P5 stocks of each strain (GNU-2353-P1 and -P5 and GNU-2377-P1 and -P5) were also determined as described above and deposited in GenBank under accession numbers PQ201861–4.

### 2.5. Quantitative rRT-PCR

Cytokine production was quantified in PRRSV-infected PAM cells using rRT-PCR, as described in previous studies [17, 29, 37]. PAM-KNU cells were inoculated with each P5 virus at an MOI of 1.0 or mock infected with the culture supernatants prepared by freeze–thawing uninfected PAM-KNU cells. Total RNA was extracted from the lysates of infected cells at 24 and 48 hpi using Patho Gene-spin DNA/RNA Extraction Kit (iNtRON Biotechnology, Seongnam, South Korea) and treated with DNase I (TaKaRa, Otsu, Japan). Quantitative rRT-PCR was then performed with One Step TB Green PrimeScript RT-PCR Kit (TaKaRa) and gene-specific primer sets using CronoSTAR 96 Real-Time System [29, 37]. Expressed transcripts encoding immune-related genes were normalized to values for porcine *β*-actin, and the relative quantities of mRNA were assessed using the 2^*−ΔΔ*Ct^ method [38]. To evaluate the transcriptional activation of target genes upon virus infection, the relative fold change of each gene was calculated and compared between virus- and mock-infected PAM-KNU cells.

### 2.6. Animal Challenge Experiments and Clinical Examinations

The animal study protocol was approved by the Institutional Animal Care and Use Committee (IACUC) of CAVAC (IACUC no. CAVAC-240527-07) and conducted at CAVAC Animal Facility. Fourteen 4-week-old piglets acquired from commercial crossbred sows (Great Yorkshire × Dutch Landrace) were purchased from a PRRSV-negative pig breeding farm. Upon arrival, all animals were tested to confirm that they were not infected with porcine viruses, such as PRRSV, porcine circovirus 2 (PCV2), and swine influenza virus (SIV), or and bacteria, such as *Actinobacillus pleuropneumoniae*, *Streptococcus suis*, *Mycoplasma hyopneumoniae*, and *Hemophilus parasuis*. The piglets were randomly allocated by weight to three experimental groups (GNU-2353, GNU-2377, and mock inoculation) and housed in separated rooms. Following a 2-day acclimation period, each animal in groups 1 and 2 was challenged with GNU-2353-P5 (*n* = 5) or GNU-2377-P5 (*n* = 5) at a dose of 3 × 10^5.0^ TCID_50_/pig intranasally (1 ml/nostril) and intramuscularly (1 ml), respectively. The sham group (*n* = 4) was administrated with an equivalent volume of cell culture medium as placebo through the same routes. After virus inoculation, clinical symptoms, including anorexia, lethargy, sneezing, coughing, labored and abdominal breathing, and skin discoloration, were monitored daily for 14 days postinoculation (DPI). Rectal temperature was recorded at 0, 1, 3, 5, 7, 10, and 14 DPI. Blood and nasal swab samples were collected on the same day and independently subjected to rRT-PCR to measure viremia and viral shedding, respectively, as outlined above. In vitro transcribed PRRSV RNA with known concentrations was produced using MEGAshortscript T7 Transcription Kit (Invitrogen) and 10-fold serially diluted to generate a standard curve, as described previously [39]. Virus titers (genomic copies/ml) in the sera and nasal swabs of all animals in each group were calculated according to this standard curve. All animals were weighed at 0 and 14 DPI to estimate the average weight gain rate. Dead piglets were necropsied at the time of death, whereas surviving animals from each group were euthanized at 14 DPI for postmortem examination. Necropsy samples from the heart, liver, spleen, lung, kidney, tonsil, and lymph node, were collected and prepared as described above to determine the levels of viral distribution using rRT-PCR. Thymuses of all animals were weighed, and the thymus/weight ratio (g/kg) was measured to quantify thymic atrophy.

### 2.7. Serology

Serum samples obtained at 0, 1, 3, 5, 7, 10, and 14 DPI were used to detect anti-PRRSV antibodies using PRRS X3 Ab Test (IDEXX Laboratories, Westbrook, ME), according to the manufacturer's instructions. A sample was considered positive for antibodies against PRRSV when the sample-to-positive (S/P) ratio was 0.4 or higher.

### 2.8. Gross and Histopathological Examination

Gross lung lesions were blindly examined and scored by two pathologists on a scale 0–100, as reported previously [40]. Lung tissue samples were collected and fixed in 10% formalin for histopathology using hematoxylin and eosin staining (Sigma–Aldrich, St. Louis, MO). Lung section scores were estimated based on the severity of interstitial pneumonia (score: 0–4), as described [40, 41]. Lung sections were further subjected to immunohistochemistry (IHC) to detect the distribution of viral antigens in the lung, according to a previously described method [42]. The number of PRRSV-positive cells in lung sections was estimated using a ranked score of 0–4, as described elsewhere [43, 44].

### 2.9. Statistical Analysis

All values are presented as the mean ± standard deviation of mean difference (SDM). Determination of statistical differences among groups was performed using a one- or two-way analysis of variance (ANOVA) with GraphPad Prism 8 software (GraphPad Software, San Diego, CA). If a *p*-value is 0.05 or lower, the result was deemed to be statistically significant.

## 3. Results

### 3.1. Genomic Characteristics of NADC30-Like GNU-2377 and NADC34-Like GNU-2353 Strains

The initial virus-specific rRT-PCR assays confirmed that the lung (farm A) and serum (farm B) specimens were tested positive for PRRSV, with Ct values of 13.50–14.69 and 16.10–19.89, respectively, but negative for other viral pathogens, including PCV2 and SIV. Subsequent amplification and sequencing of the ORF5 gene revealed that PRRSV strains GNU-2353 (farm A) and GNU-2377 (farm B), circulating in two herds, were genetically divergent from each other (11.5% aa distance) as well as from VR-2332 (>15% aa distance) (Table 1). By contrast, GNU-2353 and GNU-2377 viruses independently shared the highest similarity with NADC34-like L1A and NADC30-like L1C strains, which were previously reported in South Korea, showing 99.5% and 92.0% aa homology, respectively (Table 1).

We then deciphered the complete genomic sequences of GNU-2353 and GNU-2377 directly from the corresponding clinical samples (P0) to evaluate their genotypic traits. The full genomes of GNU-2353-P0 and GNU-2377-P0 contained 15,109 and 15,020 nucleotides (excluding the 3′-poly (A) tail), respectively, which were 303- and 392-nucleotide (nt) sequences shorter than that of PRRSV-2 prototype VR-2332 (the parent of Ingelvac MLV, L5), resulting from considerable deletions (DELs) within nsp2. The 5′-UTRs of GNU-2353-P0 and GNU-2377-P0 were 3-nt shorter (i.e., 3-nt DEL) and 1-nt longer (i.e., 2-nt insertion and 1-nt DEL) than that of VR-2332, respectively. The genomic sequences of GNU-2353 and GNU-2377 were compared with those of domestic and international reference PRRSV strains representing L1 and L5 (Table 1). Comparative WGS revealed that GNU-2353 and GNU-2377 shared 82.9% identity with each other, 85.4% and 84.9% with MN184C (L1F, formerly L1.9), 81.7%–82.5% with CA-2 and KNU-12-KJ4 (L1J, formerly LKC), and 82.7% and 81.4% with VR-2332 (L5), respectively. By contrast, GNU-2353 and GNU-2377 displayed the highest genomic similarity with representative L1A (NADC34-like, 89.4%–99.0% homology) and L1C (NADC30-like, 85.6%–92.6% homology) PRRSV-2 strains, respectively.

**Figure 1:**
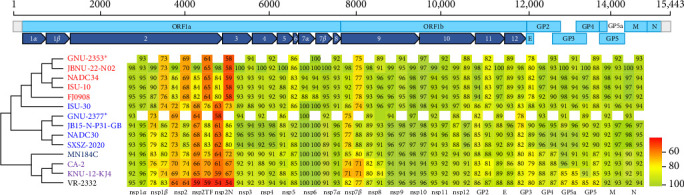
Conservation and variation of the nonstructural and structural proteins of GNU-2353 and GNU-2377 compared with those of different lineage strains of PRRSV-2. The illustration on the top depicts genomic regions, with sky blue bars representing the identified ORFs and navy blue arrows displaying the nonstructural proteins produced after translation of ORF1a/1b and subsequent processing by viral proteases. Light gray bars denote 5′- and 3′-UTRs. The whole-genome–based phylogenetic tree of lineage-representative strains is displayed on the left. Lineages of PRRSV-2 are color coded, including L1A (red), L1C (blue), L1F (navy blue), L1J (purple), and L5 (black). The newly isolated GNU-2353 and GNU-2377 strains in this study are marked with an asterisk (*⁣*^*∗*^). Heatmaps were constructed from a set of representative strains as indicated, using alignment data paired with neighbor-joining phylogenetic trees built in Geneious Prime (version 2024.0.7) and visualized with Prism 8 (version 8.4.3).

**Figure 2:**
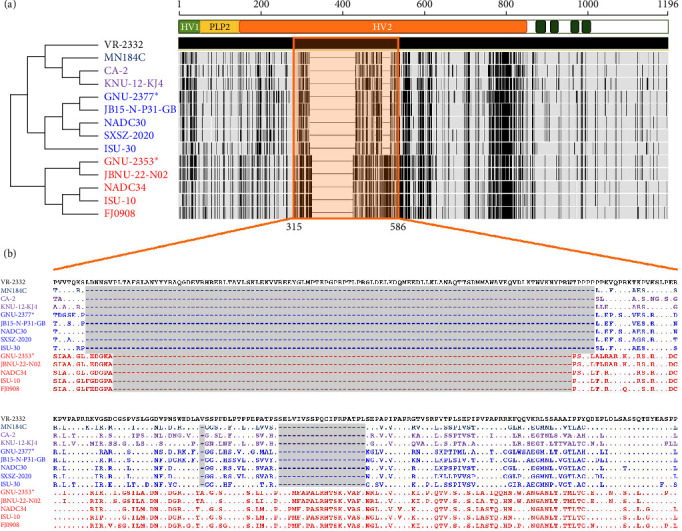
Schematic diagram of multiple alignments of nsp2 in relation to the prototype strain VR-2332 (L5). Lineages of PRRSV-2 are color coded, including L5 (VR-2232; black), L1F (navy blue), L1J (purple), L1C (blue), and L1A (red). GNU-2353 and GNU-2377 are marked with an asterisk (*⁣*^*∗*^). (a) Differences in the amino acid sequence of the nsp2-coding region across PRRSV strains. The illustration on the top represents the organization of the PRRSV nsp2 protein, highlighting the PLP2 enzymatic domain located between the two hypervariable regions (HVs) and the putative transmembrane regions indicated by dark green bars. The nsp2-based phylogenetic tree of lineage representative strains is displayed on the left. Schematic diagram (barcode profiles) showing alignment of the nsp2 gene compared with VR-2332 sequence was generated using Geneious Prime (version 2024.0.7). Lightly shaded areas indicate areas identical to VR-2332, and vertical black bars represent aa sequence divergent from that of VR-2332. The partial HV2 of nsp2 covering the molecular deletion (DEL) marker is shaded and outlined in vivid orange. Thin horizontal dashed lines indicate discontinuous and continuous DELs. (b) Amino acid sequence alignments of the partial HV2 of nsp2 (residues 315–586). The bottom panel presents aa sequence alignment of the HV2 regions of nsp2 across PRRSV strains. The molecular DEL signature, including 131-aa tripartite (111-1-19) discontinuous and 100-aa continuous DELs, within the PRRSV HV2 region is shaded in gray.

Although the nsps were highly conserved across PRRSV-2 strains, nsp1*β*, nsp2, nsp2TF, nsp2N, and nsp7*β* of GNU-2353 and GNU-2377 shared relatively low aa sequence identities with the reference L1F and L1J strains as well as their counterparts from the United States and China (Figure 1). Of those, nsp2 and its two truncated forms of GNU-2353 and GNU-2377 showed the largest diversity (>2%–46% aa distance) compared with the representative reference PRRSV-2 strains; nevertheless, GNU-2353 and GNU-2377 shared the highest nsp2 similarity with their corresponding L1A (NADC34-like, 83.4%–98.7% aa identity) and L1C (NADC30-like, 78.1%–89.3% aa identity) strains, respectively (Table 1). In addition, GNU-2353 and GNU-2377 retained lineage-specific nsp2 DEL markers compared with VR-2332. GNU-2353 harbored a continuous 100-aa DEL at residues 328–427 within the hypervariable region 2 (HV2) of nsp2, which was indistinguishable from the patterns in other L1A (NADC34-like) strains. GNU-2377 encompassed a tripartite discontinuous 131-aa DEL signature of 111-1-19, composed of 111-, 1-, and 19-aa DEL sequences at residues 322–432, 483, and 500–518 of nsp2, respectively, which was consistent with that reported for L1C (NADC30-like) PRRSV as well as L1F and L1J strains (Figure 2). Given the genomic data and unique nsp2 DEL signature, GNU-2353 and GNU-2377 were NADC34-like (L1A) and NADC30-like (L1C) strains circulating in South Korea, respectively.

**Figure 3:**
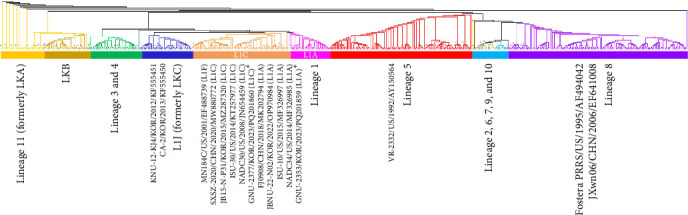
Phylogenetic analysis based on the nucleotide sequences of ORF5 of PRRSV-2 strains. Multiple sequence alignments were performed using ClustalX. Phylogenetic trees were constructed with the aligned nucleotide sequences using the neighbor-joining method. Eleven monophyletic lineages (L1–L11) of PRRSV-2 are indicated using different colors: L8 (purple), L5 (red), L3 and L4 (green), undefined nation-specific LKB (dark goldenrod), and L11 (amber). Global sublineages of L1, including NADC34-like L1A (pink), NADC30-like L1C (orange), and L1J (blue), are also indicated. GNU-2353 and GNU-2377 are marked with an asterisk (*⁣*^*∗*^). The names of the isolates, countries along with the dates (year) of isolation, and GenBank accession numbers of lineage-representative strains are included.

ORF2a–ORF7 encode various structural proteins that show a high degree of sequence variation across PRRSV-2 isolates. Notably, the glycoproteins were the most vulnerable to genetic drift among structural proteins, contributing to hypervariability (Figure 1). GP3 of GNU-2353 and GNU-2377 shared the lowest sequence identity across corresponding lineage isolates: aa identity with representative NADC34-like (L1A) and NADC30-like (L1C) strains was 89.0%–97.6% and 88.2%–90.2%, respectively (Table 1). ORF5, which encodes a major glycoprotein GP5, is another mutation hotspot in the PRRSV genome and contributes to the virological and immunological significance and polymorphic traits of PRRSV. Therefore, this region is used for restriction fragment length polymorphism (RFLP) and phylogenetic classification. The GP5 protein of GNU-2353 and GNU-2377 shared 95.0%–99.5% and 88.0%–92.0% aa identity with L1A and L1C strains, respectively, and 81.5%–90.5% aa identity with the reference strains representing L1F (MN184C), L1J (CA-2 and KNU-12-KJ4), and L5 (VR-2332) (Table 1). RFLP typing revealed that GNU-2353 had a 1-6-4 RFLP pattern, which was identical to a domestic L1A strain (JBNU-22-N02) but different from international L1A strains (NADC34, ISU-10, and FJ0908) with RFLP 1-7-4. By contrast, the RFLP pattern (1-4-2) of GNU-2377 was consistent with that of representative US (ISU-30) and Chinese (SXSZ-2020) L1C strains but inconsistent with NADC30 (1-4-4) and domestic JB15-N-P31-GB (1-8-4) strains. Subsequent phylogenetic analysis based on GP5 sequence delineated PRRSV-2 into 11 lineages (L1–11), including L1J (formerly LKC) and L11 (formerly LKA), with an undefined KOR-specific cluster LKB (Figure 3). In the phylogenetic tree, GNU-2353 and GNU-2377 were grouped into L1, clustering with NADC34-like (L1A) and NADC30-like (L1C) strains, respectively. This phylogeny confirmed the aforementioned findings of comparative genome analysis, including nsp2 DEL patterns.

### 3.2. Recombination Analysis

**Figure 4:**
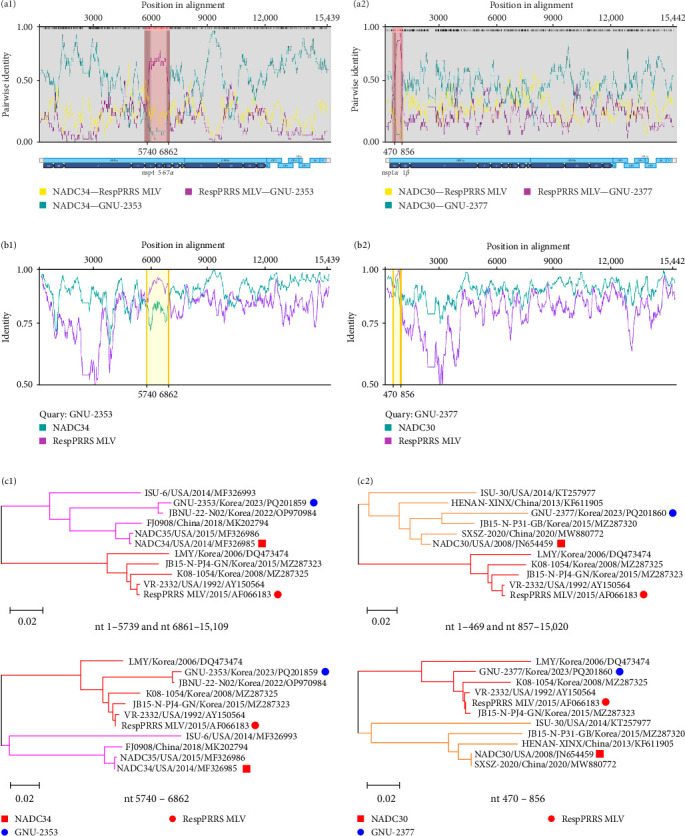
Recombination analyses of GNU-2353 and GNU-2377. (a) Recombination detection. The *x*-axis indicates genomic positions. The *y*-axis represents pairwise identity between GNU-2353 and NADC34, GNU-2353 and RespPRRS MLV, or NADC34 and RespPRRS MLV (a1) and between GNU-2377 and NADC30, GNU-2377 and RespPRRS MLV, or NADC30 and RespPRRS MLV (a2), illustrated using green, purple, and yellow lines, respectively. The start and end of each recombinant region are shaded in red and labeled with position numbers. (b) Similarity plot of GNU-2353 (b1) compared with NADC34 (green) and RespPRRS MLV (purple) and GNU-2377 (b2) compared with NADC30 (green) and RespPRRS MLV (purple). Yellow areas indicate recombination regions detected from nt 5740–6862 and nt 470–856, which encompass parts of nsp4, nsp5, nsp6, and nsp7*α*, and parts of nsp1*α* and nsp1*β*. (c) Phylogenetic trees of major and minor parental regions of GNU-2353 (c1) and GNU-2377 (c2). The major parental regions of GNU-2353 and GNU-2377 clustered with NADC34 and NADC30, respectively, whereas the minor parental regions of both strains closely correlated with those of RespPRRS MLV.

Our comparative WGS data indicated that GNU-2353 had higher aa identities (93.1%–100%) with a distinctly related L5 strain (VR-2332) in nsp4–nsp6 than with the most closely related NADC34-like L1A strains (87.5%–100%) (Figure 1). The NADC30-like L1C GNU-2377 strain shared the second highest aa similarity (82.8%) with VR-2332 (L5) for nsp1*β*. Given these results, we hypothesize that recombination occurred in the genomes of GNU-2353 and GNU-2377. To test this hypothesis, we performed genetic recombination analysis using RDP4 software. All eight methods in the RDP4 platform were utilized to determine potential recombination events and breakpoints (Figure 4a), and the data are summarized in Table 2. GNU-2353 and GNU-2377 were identified as recombinant strains, with recombination breakpoints located in nsp4 (nt 5740) and nsp6 (nt 6862) and nsp1*α* (nt 470) and nsp1*β* (nt 856), respectively (Figure 4a). The recombination events were independently corroborated by six or seven different modules with a high degree of confidence (average *p*-values, 2.749 × 10^−13^ and 2.489 × 10^−08^ for GNU-2353 and GNU-2377, respectively). NADC34 (L1A, major parent) or NADC30 (L1C, major parent) and RespPRRS MLV (L5, minor parent) were identified as the backbone and inserted sequences, respectively. Subsequent similarity comparisons confirmed that both isolates emerged from natural interlineage recombination events between L1A or L1C and L5 (RespPRRS MLV) strains (Figure 4b). SimPlot analysis proved two putative recombination breakpoints for GNU-2353 and GNU-2377. These outcomes indicate that the nsp4–nsp6 (nt 5740–6862) or nsp1*α*–nsp1*β* (nt 470–856) region of the RespPRRS MLV strain was independently introduced into the backbone of parental NADC34- and NADC30-like strains. Finally, interlineage recombinants were verified using phylogenies based on each sequence region partitioned by recombination breakpoints (Figure 4c). In the phylogenetic tree, the major parental regions of GNU-2353 and GNU-2377 outside the breakpoints (nt 1–5739 and nt 6861–15,109 or nt 1–469 and nt 857–15,020) independently clustered with NADC34 and NADC30, respectively, whereas their minor parental regions between the breakpoints (nt 5740–6862 or nt 470–856) were phylogenetically close to the RespPRRS MLV strain. Collectively, our data demonstrated that GNU-2353 and GNU-2377 are interlineage recombinants that arose through recombination between NADC34-like or NADC30-like and RespPRRS MLV viruses, respectively.

### 3.3. Virus Isolation and Cytokine Production Profile

**Figure 5:**
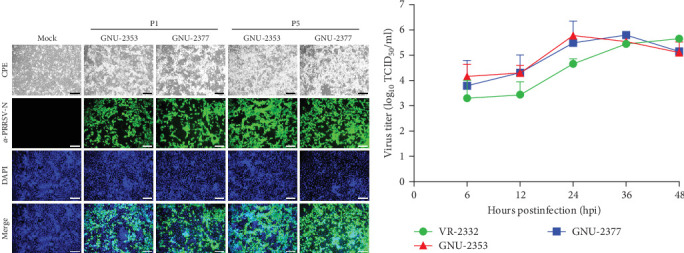
Virological properties of GNU-2353 and GNU-2377 in vitro. (a) Phenotypic characteristics of GNU-2353 and GNU-2377. PAM-KNU cells were mock infected or infected with each P1 and P5 virus at an MOI of 1.0. PRRSV-specific CPE was monitored daily, and cells were photographed at 24 hpi using an inverted microscope at a magnification of 200× (top panels). For immunostaining, infected cells were fixed at 24 hpi and incubated with an MAb against PRRSV N, followed by incubation with Alexa green-conjugated goat anti-mouse secondary antibody (second panels). The cells were nuclear stained with DAPI (third panels) and examined under a fluorescence microscope at 200× magnification (scale bar = 100 μm). (b) Growth kinetics of GNU-2353 and GNU-2377. PAM-KNU cells were inoculated with each virus strain. At the indicated time points, culture supernatants were harvested, and virus titers were determined. The results are expressed as the mean of three independent experiments performed in duplicate. Error bars indicate SDM.

We inoculated the immortalized PAM-KNU cells with PRRSV-confirmed clinical samples for virus isolation. NADC34-like GNU-2353 and NADC30-like GNU-2377 were individually isolated from each filtrate of specimens collected from the two farms and propagated in cell culture. After CPE observation was complete, the culture supernatants (P1) were passaged once in PAM-KNU cells. Each P2 strain was further cloned through three rounds of plaque purification to obtain the purified strains (GNU-2353-P5 and GNU-2377-P5). Viral growth was measured by monitoring CPE and confirmed by IFA using the PRRSV N-specific MAb (Figure 5a). In infected PAM-KNU cells, viruses from P1 and P5 generated well-defined CPE typical of PRRSV infection, such as cell rounding, clumping, and detaching, as presented in previous studies [17, 36]. CPE in PAM-KNU cells infected with GNU-2353 and GNU-2377 was noticeable at 12 hpi and became more pronounced by 24 hpi. This rate was faster than that in lab-adapted VR-2332-infected PAM-KNU cells. The specificity of PRRSV propagation was further evidenced by PRRSV-N-specific IFA, with cytoplasmic staining observed in virus-infected cells. By contrast, neither CPE nor N-specific staining was visible in mock-infected PAM-KNU cells. Examples of CPE and IFA images from selected passages are displayed in Figure 5a. The infectious titer of each isolate was ~10^4^ TCID_50_/ml at P1 and 10^6^ TCID_50_/ml at subsequent passages. Analysis of viral replication kinetics revealed that both P5 viruses (GNU-2353-P5 and GNU-2377-P5) showed indistinguishable growth curves and reached maximum titers of >10^6^ TCID_50_/ml at 24 hpi, which declined gradually (Figure 5b). By contrast, the growth curve of VR-2332 steadily increased up to 48 hpi and peaked at 48 hpi.

Complete genome sequences of the P1 and P5 viruses passaged in PAM-KNU cells were examined using WGS to evaluate whether genetic drift and recombination identified in GNU-2353-P0 and GNU-2377-P0 were conserved in cell culture. The P0, P1, and P5 sequences of GNU-2353 and GNU-2377 were compared with each other, and the differences were condensed in Table 3. Compared with the original GNU-2353-P0 virus, the P1 isolate of GNU-2353 developed one synonymous and two nonsynonymous mutations in nsp2, two synonymous mutations in nsp3, and three synonymous and three nonsynonymous mutations in nsp4, which were conserved up to passage 5. GNU-2353-P5 had one nonsynonymous substitution each in nsp11, ORF2, and ORF3 and one synonymous substitution in ORF2. During cell culture adaptation, eight aa changes emerged in the genome of NADC34-like GNU-2353, which were distributed in nsp2 (2), nsp4 (3), nsp11 (1), ORF2 (1), and ORF3 (1). By contrast, GNU-2377-P1 had five synonymous and one nonsynonymous variations in nsp2 and one nonsynonymous variation in nsp3, compared with the original P0 isolate. In addition to these changes, GNU-2377-P5 possessed two synonymous and two nonsynonymous substitutions in nsp2 and three synonymous substitutions in nsp4. During serial passages of NADC30-like GNU-2377, four nonsynonymous mutations arose—three in nsp2 and one in nsp3 (1).

**Figure 6:**
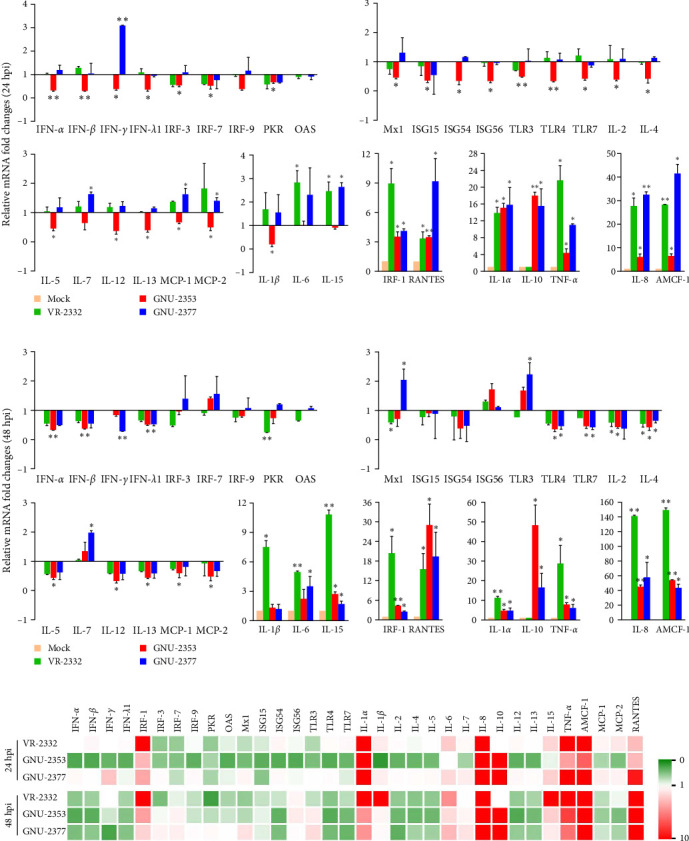
Transcriptional profiles of immune response-related genes upon PRRSV infection in vitro. (a, b) Cytokine production in PAM-KNU cells infected with each PRRSV strain at 24 (a) and 48 (b) hpi. The transcriptional level of each gene was assessed by rRT-PCR and normalized to the expression of porcine *β*-actin. Relative quantities were evaluated using the 2^*−ΔΔ*Ct^ method, and relative fold-change of each cytokine, chemokine, TLR, and antiviral gene was calculated to compare the results of virus- vs. mock-infected cells. (c) Heatmap of 34 differentially expressed immune genes in PRRSV-infected PAM-KNU cells. Heatmaps were created based on the expression for each gene with triplicates examined. Genes that were upregulated in PRRSV-infected PAM-KNU cells compared with those in mock-infected cells are displayed in red, while genes that were downregulated in PRRSV-infected PAM-KNU cells compared with those in mock infection are shown in green. Data are representative of the mean from three independent experiments, each performed in duplicate. Error bars indicate mean ± SDM. *⁣*^*∗*^*p*=0.001–0.05; *⁣*^*∗∗*^*p* < 0.001.

We next aimed to explore the effect of NADC34-like GNU-2353 or NADC30-like GNU-2377 on the transcriptional production of innate immune-related genes in the primary target cells of PRRSV. Therefore, the expression of inflammatory cytokines, chemokines, and antiviral genes in PAM-KNU cells infected with each strain was quantified using rRT-PCR and compared with that in response to VR-2232 infection. Upon infection, the transcriptional diversity of immune-related genes in PAM-KNU cells was strain dependent (Figure 6). The levels of type I interferon (IFN-*α*/*β*) mRNA were unaffected in VR-2332- and GNU-2377-PAM-KNU cells at any time point, but they continuously decreased in response to GNU-2353 during the course of infection. Although VR-2332 infection failed to elicit the production of type II IFN (IFN-*γ*) mRNA, IFN-*γ* expression varied depending on GNU-2353 and GNU-2377. A significant decline of IFN-*γ* transcript was observed in GNU-2353-infected PAM-KNU cells at 24 hpi. However, IFN-*γ* transcription was up- and downregulated in GNU-2377-infected cells, 24 and 48 hpi, respectively. No significant regulation of type III IFN (IFN-*λ*) transcription was detected in VR-2332-infected PAM-KNU cells infected with at any time point. By contrast, at 24 and/or 48 hpi, infection with GNU-2353 and GNU-2377 transcriptionally downregulated IFN-*λ* expression in PAM-KNU cells. The expression of IFN-response genes, including IFN-regulatory factor (IRF)-3 and -7; protein kinase R (PKR); Mx1; and IFN-stimulated gene-15, -54, and -56, was statistically downregulated after GNU-2353 infection and unaffected after infection with VR-2332 and GNU-2377 at 24 hpi; however, the levels of certain transcripts, including PKR and Mx1, altered at 48 hpi. Interestingly, infection with GNU-2353 and GNU-2377 significantly increased in IRF-1 expression, although not as high as that in response to VR-2332 infection. The transcription of toll-like receptor (TLR) genes remained steady in VR-2332-infected PAM-KNU cells. By contrast, infection with GNU-2353 and GNU-2377 induced a significant decrease in TLR expression, although TLR3 was slightly promoted in cells infected with either virus at 48 hpi.

Transcriptional downregulation of interleukin (IL) genes, including IL-1*β*, IL-2, IL-4, IL-5, IL-12, and IL-13, was observed in PAM-KNU cells infected with GNU-2353; by contrast, upregulation of IL-6, IL-7, and IL-15 transcripts was found in cells infected with GNU-2377 at 24 or 48 hpi. Although transcription of IL-1*α*, IL-1*β*, IL-6, and IL-15 genes was substantially upregulated, overall cytokine responses to infection with VR-2332 were insignificant. High levels of IL-1*α* and IL-8 mRNAs were detected in PAM-KNU cells infected with each of the three virus strains. In particular, no expression of IL-10 was seen upon VR-2332 infection, whereas dramatic transcriptional upregulation of IL-10 was evidenced in GNU-2353- and GNU-2377-infected PAM-KNU cells throughout the infection period, and IL-10 expression was more robust in GNU-2353-infected cells at 48 hpi. Conversely, individual strains elicited significant upregulation of TNF-*α* expression in infected PAM-KNU cells, although VR-2332 infection upregulated TNF-*α* expression more remarkably. The transcription of some chemokine genes, such as monocyte chemoattractant protein-1 (MCP-1) and MCP-2, was downregulated in GNU-2353-infected cells infected, whereas in GNU-2377-infected cells, their expression was enhanced at 24 hpi. Elevated expression of regulated upon activation, normal T cell expressed and presumably secreted (RANTES) and alveolar macrophage chemotactic factor-1 (AMCF-1) was detected in PAM-KNU cells infected with each of the three virus strains.

### 3.4. Pathogenicity Analysis of NADC30-Like GNU-2377 and NADC34-Like GNU-2353 Strains

**Figure 7:**
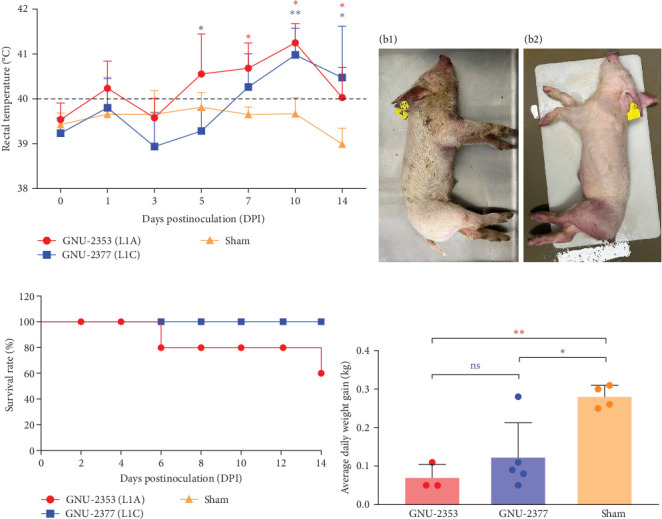
Pathogenicity results in piglets. (a) Rectal temperatures of each group. Rectal temperatures of >40°C (dashed line) were defined as high fever. (b) Clinical cyanosis observed in animals infected with GNU-2353 (b1) and GNU-2377 (b2). (c) Survival curves. Individual piglets were monitored, and survival rates of each group until 14 DPI were plotted. (d) Average daily weight gain (ADWG) of each group. ADWG values of individual pigs in each group are presented. Error bars indicate SDM. Asterisks of different colors indicate significant differences between GNU-2353 and GNU-2377 groups (purple), GNU-2353 and sham groups (red), or GNU-2377 and sham groups (blue). ns, no significant difference; *⁣*^*∗*^*p* < 0.05; *⁣*^*∗∗*^*p* < 0.01.

Because both NADC30- and NADC34-like strains were independently isolated from conventional swine herds with high mortality, we assessed their pathotypic traits. To explore the pathogenicity of GNU-2353 and GNU-2377, 14 piglets (4-week-old) were experimentally challenged. The piglets were assigned into three groups: group 1 (*n* = 5) was challenged with GNU-2353, group 2 (*n* = 5) was challenged with GNU-2377, and sham group (*n* = 4) received the cell culture medium. During acclimatization, all pigs were healthy and did not develop clinical symptoms, and their PRRSV-negative status was confirmed. Following the challenge, all piglets in the sham group presented no obvious clinical manifestations and mortality and maintained normal rectal temperatures throughout the experiment (Figure 7a). However, GNU-2353-infected piglets (group 1) displayed apparent clinical disorders starting at 2 DPI, with lethargy, inappetence, coughing, and labored breathing. In addition, GNU-2353-infected animals developed high fever at 1 DPI (average 40.2°C) and remained hyperthermic from 5 DPI until the end of the study, with a peak mean rectal temperature of 41.3°C at 10 DPI (Figure 7a). Cyanotic ears, limbs, snouts, abdomens, and vulva were concomitantly observed in some piglets (2/5) challenged with GNU-2353 (Figure 7b, b1). Although the onset of clinical manifestations was suspended in GNU-2377-infected animals in group 2, they began showing clinical signs comparable with those observed in the GNU-2353-infected group by 6 DPI. Consistently, the piglets in group 2 underwent persistent hyperthermia from 7 DPI, with a peak mean rectal temperature of 41.0°C at 10 DPI (Figure 7a). In comparison to the GNU-2377 group, GNU-2353-infected animals exhibited significantly elevated rectal temperatures at 5 DPI (Figure 7a). Similar to the GNU-2353 group, one piglet in the GNU-2377 group had severe cyanosis of the extremities (Figure 7b, b2). Notably, two piglets (40%) showing apparent cyanosis in the GNU-2353 group died at 6 and 14 DPI, respectively, whereas all piglets in the GNU-2377 group survived until trial termination (Figure 7c). As presented in Figure 7d, piglets in the GNU-2353, GNU-2377, and sham groups had average daily weight gain (ADWG) of 0.07, 0.12, and 0.28 kg, respectively. Compared to the control group, all infected groups displayed a significant decrease in ADWG, albeit the decline in ADWG was more remarkable in the GNU-2353 group (Figure 7d).

**Figure 8:**
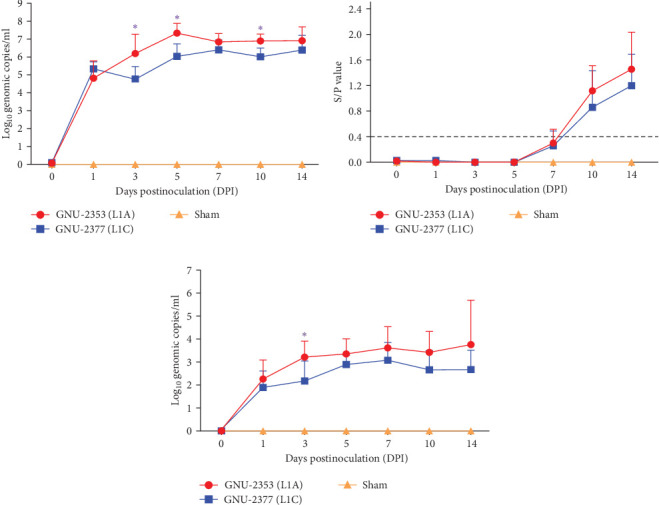
Viremia, serology, and nasal shedding in different experimental groups. (a) Viremia in each group. PRRSV RNA titers (log_10_ genomic copies/ml) in the serum of inoculated pigs at each time point were determined using rRT-PCR. (b) PRRSV-specific antibody level. The serum of each challenged piglet was assayed for PRRSV-specific antibodies on different days postinoculation. The cutoff value for seroconversion was set at a sample-to-positive (S/P) ratio of 0.4. (c) PRRSV shedding in nasal secretions. PRRSV RNA titers (log_10_ genomic copies/ml) in nasal swabs at the indicated sampling points were determined using rRT-PCR. The mean values of each group at each time point are presented. Error bars indicate SDM. *p* values were calculated by comparing data between GNU-2353- and GNU-2377-infected groups. *⁣*^*∗*^*p* < 0.05.

Blood samples were collected at 0, 1, 3, 5, 7, 10, and 14 DPI from each group to quantify viremia and serology in piglets. Viremia was detected in all piglets inoculated with GNU-2353 and GNU-2377 from 1 DPI and persisted until the end of the experiment (Figure 8a). Mean titers of the GNU-2353 and GNU-2377 groups reached peak levels of 10^7.33^ and 10^6.40^ genomic copies/ml at 5 and 7 DPI, respectively. Intriguingly, the mean serum viral loads were significantly higher in the GNU-2353 group than in the GNU-2377 group at 3, 5, and 10 DPI. Piglets in the sham group stayed vigorous with no viremia throughout the study. PRRSV-specific antibodies were measured using a commercial ELISA kit. In total, one-fifth animals in the GNU-2353 group seroconverted (S/P ratio >0.4) at 7 DPI, and all became seropositive by 10 DPI (Figure 8b). In the GNU-2377 group, one-fifth and two-fifths piglets seroconverted at 7 and 10 DPI, respectively, and the remaining two piglets were seropositive at 14 DPI. All animals in the sham group remained seronegative during the trial.

To examine PRRSV shedding in each group, viral RNA was quantified in nasal secretions. The kinetics of viral shedding in the respiratory tract were different between the GNU-2353- and GNU-2377-inoculated groups (Figure 8c). In the GNU-2353 group, nasal shedding started in all animals at 1 DPI, with a mean titer of 10^2.26^ genomic copies/ml, and the amount of viral load steadily increased and reached a peak level of 10^3.76^ genomic copies/ml at 14 DPI. Similarly, all piglets in the GNU-2377 group shed viruses in their nasal secretions by 1 DPI, with a mean titer of 10^1.90^ genomic copies/ml. However, viral shedding through the nasal route reached a peak titer of 10^3.09^ genomic copies/ml at 7 DPI and decreased until 14 DPI. There was a significant difference in the quantity of viral shedding between the GNU-2353 and GNU-2377 groups at 3 DPI. In the sham group, nasal samples remained vironegative throughout the study.

**Figure 9:**
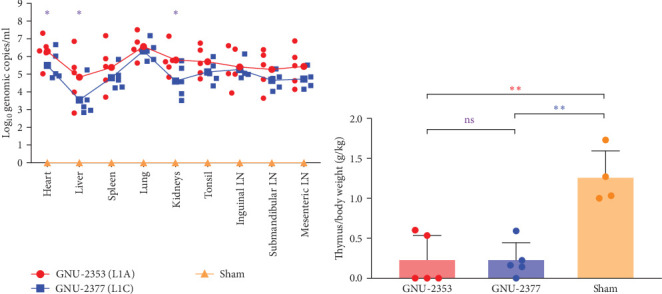
Virus tissue distribution and thymus/body weight gain in piglets. (a) PRRSV distribution in various tissues. PRRSV RNA load in each tissue collected from individual piglets at necropsy (performed upon death or after euthanasia at 14 DPI) was determined using rRT-PCR. Virus titers were expressed as genomic copies/ml. Error bars indicate SDM. *p* values were calculated by comparing results from GNU-2353- and GNU-2377-infected groups. (b) Thymic atrophy in PRRSV-infected piglets. Thymus/body weight at 14 DPI in the different experimental groups. Thymic atrophy was quantified as the ratio of the thymus to total body weight (g/kg). Mean ± SD (error bars) of the thymus/body weight ratio is shown. Asterisks of different colors indicate significant differences between GNU-2353- and GNU-2377-infected groups (purple), GNU-2353-infected and sham groups (red), or GNU-2377-infected and sham groups (blue). ns, no significant difference; *⁣*^*∗*^*p* < 0.05; *⁣*^*∗∗*^*p* < 0.01.

PRRSV RNA loads were determined in various tissues collected during necropsy. The measurement of tissue viral loads revealed that both GNU-2353 and GNU-2377 viruses were widely distributed in all collected tissues of every infected piglet (Figure 9a). As expected, the lungs of all infected piglets contained the highest viral load, which was comparable between the GNU-2353- and 2377-challenged groups, with mean titers of 10^6.56^ and 10^6.32^ genomic copies/ml, respectively. However, mean viral loads in other organs were significantly higher in the GNU-2353 group than in the GNU-2377 group, particularly in the heart (10^6.30^ genomic copies/ml), liver (10^4.83^ genomic copies/ml), and kidney (10^5.79^ genomic copies/ml). By contrast, PRRSV RNA remained undetectable in all samples collected from the sham group.

All surviving animals were euthanized and necropsied after trial termination for postmortem assessment; however, two piglets in the GNU-2353 group that died at 6 and 14 DPI were necropsied upon death. Because highly pathogenic PRRSV (HP-PRRSV) induced severe thymic atrophy [45, 46], we evaluated the pathogenicity of PRRSV by quantifying the degree of thymic atrophy. To accomplish this, the average ratio of thymus/body weight (g/kg) was measured for each group. In the sham group, no animal presented with obvious thymus atrophy, and the average ratio of thymus/body weight was 1.26 g/kg (Figure 9b). However, in the GNU-2353 and GNU-2377 groups, a significant alteration in the average ratio of thymus/body weight, representing thymus atrophy, was observed (0.23 and 0.22 g/kg, respectively), compared with the control group.

**Figure 10:**
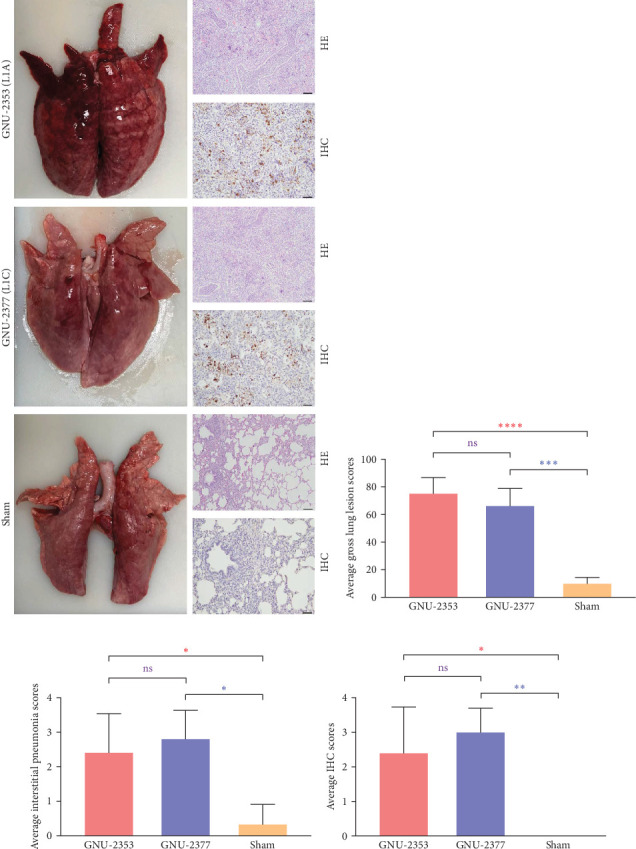
Macroscopic and microscopic lung lesions in piglets. (a) Gross, histopathological, and immunohistochemistry (IHC) examinations. Lungs of individual piglets from each group were examined for gross lesions. Representative necropsy images are presented on the left of each panel. Note that piglets infected with GNU-2353 and GNU-2377 presented multifocal pulmonary lesions. No gross lung changes were observed in sham-infected piglets. Hematoxylin and eosin–stained lung tissue sections from representative piglets in each group are shown at the upper right of each panel (100× magnification, scale bar = 100 μm). Interstitial pneumonia with necrotic inflammatory cell infiltration in the alveoli is seen in the lung sections of GNU-2353 and GNU-2377-inoculated piglets. No microscopic lesions were found in the sham group. IHC analysis results showing PRRSV antigen in lung sections from representative piglets in each group are presented at the bottom right of each panel (200× magnification, scale bar = 50 μm). IHC staining of PRRSV N was detected in the lung sections of piglets inoculated with GNU-2353 and GNU-2377. No PRRSV antigen was identified in the lung of sham-inoculated piglets. (b–d) Scores of average gross lung lesions (b), interstitial pneumonia (c), and IHC (d) in the challenged piglets. The mean values of each group are presented. Error bars indicate SDM. Asterisks of different colors indicate significant differences between GNU-2353-infected and GNU-2377-infected groups (purple), GNU-2353-infected and sham groups (red), or GNU-2377-infected and sham groups (blue). ns, no significant difference; *⁣*^*∗*^*p*  < 0.05; *⁣*^*∗∗*^*p*  < 0.01; *⁣*^*∗∗∗*^*p*  < 0.001; *⁣*^*∗∗∗∗*^*p*  < 0.0001.

At necropsy, GNU-2353- and GNU-2377-infected animals macroscopically displayed multifocal pulmonary lesions characterized by interstitial pneumonia and reddish lung consolidation with hemorrhagic spots, together with hemorrhage in the tonsils and enlarged lymph nodes, whereas no visible lung lesions were noted in control animals (Figure 10a). The two challenged groups had no significant difference in average gross lung lesion scores; however, both groups had significantly higher average gross lung lesion scores than the sham group (Figure 10b). Subsequent histopathological examination confirmed that microscopic lung lesions were similar between the GNU-2353- and GNU-2377-infected groups, but the severity varied from mild to severe (Figure 10a). Animals inoculated with GNU-2353 and GNU-2377 showed mild to severe interstitial pneumonia with necrotic inflammatory cell infiltration in the alveoli. The presence of PRRSV antigens in the lungs of pigs in each group was further examined using IHC. As presented in Figure 10a, PRRSV-positive antigens were markedly detected in the lungs of all animals in groups 1 and 2. Neither microscopic lung changes nor IHC-stained cells were seen in the sham group (Figure 10a). Consistent with the findings of histopathological and IHC assessments, mean microscopic lung lesion and IHC scores of the GNU-2353 and GNU-2377 groups were comparable but significantly higher than those of the sham group (Figures 10c and 10d).

## 4. Discussion

Notwithstanding the nationwide usage of multiple MLV vaccines in South Korea, PRRSV has been scarcely controlled and is widely disseminated in swine herds across the country. Therefore, PRRSV has caused great socioeconomic damage to the domestic pork industry since its first detection in the mid-1980s in South Korea [47]. Because this virus undergoes high-frequency genetic drift owing to error-prone viral RNA polymerase and is greatly susceptible to natural recombination [48, 49], continuous spatial and temporal evolution of PRRSV has occurred in countries endemic to PRRSV. This has triggered extreme genetic and antigenic diversity and led to the advent of PRRSV mutants with novel pathotypes and immune escape or the concomitant existence of numerous heterologous strains, which make the control of PRRSV in the field challenging [50]. Accordingly, PRRSV-2 has been highly prevalent, along with the emergence of local PRRSV-2 variants, in South Korea, albeit PRRSV-1 has existed singly or with PRRSV-2 in the domestic pig population [15]. At present, the main epidemic strains of PRRSV-2 can be further classified into four (sub)lineages, including L1, L5, LKC (reclassified as sublineage L1J), and LKB (undefined KOR-specific lineage) [10, 15]. The occurrence and circulation of L1 populations, particularly virulent sublineages L1A (NADC34-like PRRSV) and L1C (NADC30-like PRRSV), continue to be reported worldwide and are accountable for regional epidemics with explosive abortions in sows and high mortality in piglets in Asia and North America [24–26, 51–53]. Similarly, L1 of PRRSV has become the most dominant lineage in South Korea since the 2020s [15]. Since then the incidence of infection from the virulent sublineages L1A and L1C viruses has increased nationwide, causing unprecedented reproductive and productive loss to the domestic pig industry [15, 27]. Despite their devastating outcomes in the field, studies on the genetic diversity and pathogenicity of NADC30-like (L1C) and NADC34-like (L1A) PRRSV issuing in South Korea have been limited.

In this study, two novel PRRSV isolates (GNU-2353 and GNU-2377) were identified in clinical samples collected from growing piglets in two Ingelvac MLV-vaccinated farms with increased postweaning mortality. Preliminary sequencing of ORF5 revealed that the GNU-2353 and GNU-2377 viruses circulating in the farms were genetically close to NADC34-like (L1A) and NADC30-like (L1C) strains reported globally. Subsequent WGS analysis confirmed the ORF5 comparison data, showing the highest nt identity with respective L1A and L1C reference strains at the whole-genome level. The nsp2-coding region possesses the exclusive DEL marker that can verify specific (sub)lineages of PRRSV-2, such as a 30-aa discontinuous DEL for L8 (HP-PRRSV-like); a 131-aa discontinuous DEL for L1F (MN184-like), L1C (NADC30-like), and L1J (LKC-like); and a 100-aa continuous DEL for L1A (NADC34-like) [17, 24, 54, 55]. Consistent with other reports, GNU-2353 and GNU-2377 maintained L1A- and L1C-specific molecular signatures that harbor 100-aa continuous and 131-aa discontinuous DELs in each nsp2, respectively. In the ORF5-based phylogenetic tree, GNU-2353 and GNU-2377 were classified into L1A and L1C, respectively, grouped separately with their genetically corresponding relatives. In most cases, ORF5 RFLP typing alone cannot authentically reflect the genetic relatedness and diversity of PRRSV-2 strains [10]. Our study presented inconsistent results from RFLP and phylogenetic classification, showing 1-6-4 and 1-4-2 RFLP patterns for GNU-2353 and GNU-2377, which were dissimilar to classical 1-7-4 and 1-4-4 categories of NADC34- and NADC30-like PRRSV, respectively. Nevertheless, considering all genomic and phylogenetic data, the GNU-2353 and GNU-2377 strains isolated in this study are NADC34-like (L1A) and NADC30-like (L1C) viruses, respectively, that adversely affect pig herds in South Korea.

Recombination—a vigorous driving force for PRRSV evolution, in addition to genetic drift, has played an important role in the emergence of PRRSV variant strains with new genotypes and pathotypes that can be resistant to immunization [52, 56]. Moreover, field situations that simultaneously comingle heterogeneous lineages of PRRSV-2 provide comfortable surroundings for viral recombination. Indeed, numerous biparental recombination events between NADC30-like (L1C) or NADC34-like (L1A) PRRSV and local epidemic strains (e.g., L3, L5, and L8) or between NADC30- and NADC34-like strains have been frequently observed in many countries, including the United States [57, 58], China [52, 59–65], and South Korea [27, 66]. Triparental and quadriparental recombination between three (L1C, L5, and L8; L1C, L8, and L3; and L1A, L1C, and L8) or four lineages (L1C, L5, L8, and L3) of PRRSV-2 has been reported [43, 56, 67, 68]. In the current study, genome alignments found that ORFs 2–7 of GNU-2353 and GNU-2377 shared 94.2%–95.6% and 91.1%–94.1% nt homology with their corresponding lineage reference strains, respectively; however, their ORF1a and ORF1b exhibited relatively lower similarities than ORFs 2–7 (Table 1). Attractively, GNU-2353 and GNU-2377 genetically resembled VR-2332 (L5) in the nsp4–nsp6 and nsp1*α* coding regions, respectively, rather than NADC34- and NADC30-like strains, suggesting a recombination event. Recombination and phylogenetic analyses confirmed that the GNU-2353 and GNU-2377 viruses individually stemmed from biparental recombination between two lineages, L1A (NADC34-like) and L5 (RespPRRS MLV-like) or L1C (NADC30-like) and L5 (RespPRRS MLV-like), with recombination breakpoints in nsp4–nsp7*α* (nt 5740–6862) and nsp1*α*–nsp1*β* (nt 470–856), respectively. Although the recombination pattern of NADC34-like strains prevalent in South Korea has been recently described [27], GNU-2377 is the first report on the recombination profile between NADC30-like (L1C) and RespPRRS MLV-like (L5) viruses. Corresponding to our findings, a recent study reported that biparental recombination between circulating PRRSV lineages and RespPRRS MLV is the most frequent recombination profile in South Kora [66]. Furthermore, the Ingelvac MLV-derived L5 is one of the most popular PRRSV-2 lineages in South Korea, likely resulting from unwarranted mass immunization with Ingelvac MLV, which has been most commonly used for almost three decades in the field [13, 15, 16]. Importantly, the extensive use of several MLV vaccines against PRRSV-1 and PRRSV-2 in South Korea may raise safety concerns regarding viral shedding and spread among pigs, amplifying the risk of recombination with coinfected other circulating strains. Given these challenges, genetic recombination between epidemic and vaccine-related lineages might be inevitable, and thus, it is necessary to monitor and surveil the emergence and outbreak of such recombinants.

Because the MARC-145 cell line was nonpermissive to many PRRSV field strains, including NADC30- and NADC34-like viruses [52, 56, 64, 69], this study employed the immortalized PAM-KNU cell line that has been solely used in our laboratory for PRRSV isolation and research purposes [17, 36, 70, 71]. The NADC34-like GNU-2353 and NADC30-like GNU-2377 strains were isolated successfully and replicated in PAM-KNU cells. PRRSV growth kinetics revealed that GNU-2353 and GNU-2377 had better growth with higher titers than PAM-KNU-passaged VR-2332 virus at 24 hpi. The complete genome sequences of cell-passaged GNU-2353 and GNU-2377 strains were further determined. Compared to the original P0 isolates, eight and four nonsynonymous substitutions leading to aa changes arose in nsp2 (2), nsp4 (3), nsp11 (1), GP2 (1), and GP3 (1) of GNU-2353 and nsp2 (3) and nsp3 (1) of GNU-2377 during cell passage, respectively. The results of recombination detection assays using each P0 isolate were indistinguishably reproduced when the whole-genome sequence of each P5 virus was utilized. Notably, the 6 nt variations in nsp4 of GNU-2353-P5 had no effect on recombination breakpoints positioned in nsp4–nsp7*α* of GNU-2353. PAM-KNU cell culture has been used to obtain indirect in vitro evidence for PRRSV virulence by comparing the transcriptional profiles of immune-associated genes between cells infected with a field isolate and attenuated VR-2332 virus [17, 31, 32, 72]. Our study indicated that the transcriptional levels of most cytokine and chemokine genes following infection with GNU-2353 and GNU-2377 were significantly distinguishable from those after VR-2332 infection. In particular, the majority of IFN, IFN-response, and IL genes were transcriptionally repressed in GNU-2353-infected PAM-KNU cells at 24 hpi. The downregulation of innate immune-mediated cytokine gene production was probably due to significantly reduced expression of TLR genes, whose activation upon viral infection triggers signaling cascades to produce cytokine genes, such as IFNs and IFN-driven antiviral genes. Accordingly, the NADC34-like GNU-2353 strain may manipulate innate immunity early in infection, subvert the antiviral environment, and favor viral replication [17, 73]. More remarkably, the anti-inflammatory cytokine IL-10 was greatly elicited in GNU-2353- and GNU-2377-infected PAM-KNU cells. Although the role of IL-10 in PRRSV pathogenesis is debatable [74, 75], IL-10 limited host immune defense to help establish persistent PRRSV infection [76]. Consistent with a previous study, VR-2332 infection upregulated TNF-*α* transcript expression in PAM-KNU cells more potently than the field isolates [17]. Overall, our findings indicate that GNU-2353 and GNU-2377 suppressed antiviral cytokine production and promoted anti-inflammatory IL-10 expression, which may be associated with their potential to survive or spread within pigs by subverting host innate immunity.

Globally, NADC30- and NADC34-like PRRSV strains displayed broad pathogenic diversity under experimental conditions. In the United States, the NADC30 virus exhibited moderate pathogenicity with clinical manifestations of fever and low mortality [54]. In 2013, novel PRRSV strains with distinctive genetic traits frequently emerged in China and were designated NADC30-like PRRSV owing to the highest genomic similarity with the US NADC30 strains [22, 77, 78]. Recent animal challenge trials have demonstrated the virulence of Chinese NADC30-like strains that cause milder illness and lower mortality rates than HP-PRRSV isolates [26, 44, 79]. Similarly, Liu et al. [43] reported the low pathogenicity of NADC30-like PRRSV that does not cause death in piglets. By contrast, the highly virulent NADC30-like GXGG-8011 strain, which caused high mortality (80%) in challenged pigs, was reported in China. In addition, Zhang et al. [26] noted the emergence of novel PRRSV strains arising from biparental recombination between NADC30- and NADC34-like isolates in Chinese swine herds experiencing 20% abortion rates in sows and 10% lethality in piglets. On the other hand, after the first outbreak of the NADC34 virus in 2014 in the United States [23], the same lineage of PRRSV was identified in 2017 in China and named NADC34-like PRRSV due to its close genetic relatedness to the US NADC34 strain [26]. Despite the high genomic resemblance between these two viruses without any recombination signal, a discrepancy in pathogenicity has been reported in the pathogenicity between US NADC34 and Chinese NADC34-like viruses: US NADC34 exhibited high pathogenicity with prolonged hyperthermia and high mortality (>14%) [24], whereas Chinese NADC34-like had mild and moderate virulence without higher fever and death [52, 80, 81]. However, Yuan et al. [69] demonstrated the high pathogenicity of the Chinese NADC34-like JS2021 strain in pigs, with persistent fever, reduced body weight, and high mortality (>70%). In recent years, various highly virulent NADC34-like recombinants derived from bi-, tri, and quadri-parental recombinations with local epidemic lineages of PRRSV-2, including L1C (NADC30-like), L8 (HP-PRRSV-like), and/or L3, have emerged in China. The severe pathogenicity of these strains in the field is frequently represented by high abortion rates in sows and increased lethality rates in piglets [26, 65, 82]. The pathotypic feature with high mortality has been reproduced under experimental conditions in piglets [56, 60].

Given the wide-ranging variations in the virulence of international NADC30- and NADC34-like strains, it was interesting to assess the pathogenicity of the domestic strains in pigs. Kim et al. [27] reported the first NADC34-like strain identified in South Korea and expected high pathogenicity in pigs due to its replication ability and adaptation to PAMs, which may be associated with PRRSV pathogenicity and virulence [62]. In this study, in vivo experiments were conducted to examine various clinical parameters, including the duration and severity of hyperthermia and viremia, which are practically used to measure the virulence of PRRSV [83–85]. Following challenge, piglets infected with NADC34-like GNU-2353 and NADC30-like GNU-2377 strains commonly displayed pronounced clinical symptoms, high fever (>40°C), reduced ADWG, high viremia and nasal shedding, viral distribution in various tissues, and obvious interstitial pneumonia compared with control animals throughout the trial, indicating that both strains are virulent to pigs. Studies have revealed that the ability of HP-PRRSV to induce thymic atrophy is closely related to its pathogenicity [46, 61]. Consistently, our findings demonstrated that both NADC34-like GNU-2353 and NADC30-like GNU-2377 can cause severe thymic atrophy or loss in infected piglets. However, certain experimental outcomes regarding clinical severity, hyperthermia, mortality, and viral loads in the GNU-2353 group were significantly more severe than those in the GNU-2377 group. GNU-2353-infected piglets had a faster onset of apparent clinical abnormalities and more prolonged hyperthermia than GNU-2377-infected animals. Peripheral cyanosis was observed in 40% (2/5) and 20% (1/5) of the piglets infected with GNU-2353 and GNU-2377. Mortality occurred in two cyanotic piglets in the GNU-2353-infected group. GNU-2353-infected piglets had higher viremia and shed higher amounts of virus through the nasal route than GNU-2377 over the course of infection, indicating that GNU-2353 has greater replication and dissemination efficiency in piglets. Although tissue distribution of both NADC34-like GNU-2353 and NADC30-like GNU-2377 viruses was comparable, GNU-2353-infected pigs had higher viral loads in several tissues than GNU-2377-infected animals. Collectively, our work corroborates that NADC30-like GNU-2377 PRRSV exhibited significant pathogenicity in piglets but had lower virulence than NADC34-like GNU-2353 PRRSV. By contrast, recent pathogenicity studies have found that Chinese NADC34-like PRRSV is highly pathogenic but has lower pathogenicity than Chinese NADC30-like PRRSV [86]. The disparity of pathogenicity results between KOR and Chinese NADC30- and NADC34-like PRRSV could be ascribed to several factors, including experimental duration (14 days vs. 28 days), pig age (4-week old vs. 8-week old), and virus origin: Chinses NADC30- and NADC34-like viruses had no recombination with other strains, whereas GNU-2353 and GNU-2377 originated from interlineage recombination between NADC34-like (L1A) or NADC30-like (L1C) and RespPRRS MLV-like (L5) strains in the nsp-coding regions. In the present study, we could not identify major parental NADC30- and NADC34-like strains without recombination, which can enable us to investigate the virulence of nonrecombinant and recombinant viruses to ascertain the correlation between recombination and pathogenicity. Virulence studies have shown that recombination serves as PRRSV's almighty talent that cannot only generate a broad-range of novel variants but also play critical roles in adaptation and virulence in pigs [21, 60, 61, 77, 87–89]. Furthermore, accumulating evidence supports that recombination is accountable for the genotypic and pathotypic variances of NADC30- and NADC34-like PRRSV, and the pathogenicity of their recombinant variants tends to be an intermediate between those of the parental strains [53]. Alternatively, clinical observations have indicated high abortion rates on domestic farms during NADC30- and NADC34-like PRRSV outbreaks, suggesting that high virulence in gilts and sows is associated with abortion. Therefore, further, in vivo investigations are necessary to evaluate the reproductive pathogenicity of GNU-2353 and GNU-2377 in sows.

In conclusion, the increasing number of NADC34-like (L1A) and NADC30-like (L1C) PRRSV outbreaks with high abortion and mortality rates in South Korea have made L1A and L1C strains of PRRSV-2 one of the greatest concerns in the domestic swine industry. Despite tremendous interest, this topic has been under-researched, with knowledge gaps on genotypic and pathotypic characteristics of the domestic viruses devastating pig farming. The present study isolated NADC34-like GNU-2353 and NADC30-like GNU-2377 PRRSV-2 strains from two distinct Ingelvac MLV-vaccinated swine herds undergoing PRRSV-related reproductive and productive loss. Our findings indicate that GNU-2353 and GNU-2377 evolved independently through natural interlineage recombination between NADC34- or NADC30-like and RespPRRS MLV-like strains and exhibited high and moderate pathogenicity in piglets. Considering the implementation of vaccination before the incidence in each farm, Ingelvac MLV provided incomplete cross-protection against each heterologous GNU-2353 and GNU-2377 strain. Under this circumstance, the recombinant NADC34- and NADC30-like viruses appear to rapidly evolve to competently adapt to pig herds. The rapid accumulation of mutations (i.e., genetic drift) and recombination (i.e., genetic shift) has accelerated the genetic and antigenic heterogeneity and complexity of PRRSV, leading to the generation of new immune escape or pathotypic variants poised in emergence into pig populations, which hinders attempts toward controlling and eliminating PRRSV. Because MLV vaccines cannot confer universal protection against contemporary epidemic NADC34- and NADC30-like viruses, vaccination may result in vaccine failure and further immune selection pressure, which hastens viral evolution. Therefore, the collaborative endeavors to implement strict biosecurity practices, accomplish continuous surveillance, and monitoring of emerging strains, and develop safe and effective vaccines tailored to the field epidemics are important to control PRRSV and impede forthcoming outbreaks.

## Figures and Tables

**Table 1:** Detailed comparison of the whole-genome sequences of GNU-2353 and GNU-2377 and other lineage-representative PRRSV-2 strains.

Identity with GNU-2353/GNU-2377 (%)	Lineages	L1F	L1A (NADC34-like)				L1C (NADC30-like)				L1J (formerly LKC)		L5
	GNU-2353 GNU-2377	MN184C	NADC34	ISU-10	FJ0908	JBNU-22-N02	NADC30	ISU-30	SXSZ-2020	JB15-N-P31-GB	CA-2	KNU-12-KJ4	VR-2332
Nucleotides (length)													
5′-UTR (187/191)	91.1	95.3/94.3	95.7/93.8	95.2/93.2	94.7/92.7	100/90.1	93.2/94.8	88.6/88.4	91.6/93.8	92.3/93.6	91.6/89.1	90.5/89.1	94.2/89.6
ORF1a (7212/7119)	78.9	80.9/82.1	87.6/78.6	87.0/78.4	86.4/78.3	99.0/79.2	82.5/89.5	81.9/82.1	81.3/87.4	80.9/91.0	78.4/80.2	77.8/79.7	78.3/76.6
ORF1b (4377/4377)	86.3	90.7/87.9	91.0/86.5	90.8/86.3	90.5/86.3	99.1/86.5	89.1/92.8	91.7/86.9	88.3/91.8	87.7/93.8	84.6/84.0	84.2/83.7	86.4/85.9
ORF2−7 (3188/3188)	86.1	87.5/86.1	95.6/87.8	95.5/87.8	94.2/86.6	99.2/86.3	89.0/92.6	88.0/91.1	89.0/92.3	87.7/94.1	85.7/84.4	86.1/84.7	86.9/85.5
3′-UTR (151/151)	93.4	94.7/94.7	98.3/94.4	98.7/94.7	97.4/93.4	98.0/95.4	95.4/96.7	94.0/92.7	95.4/96.7	91.9/96.7	94.7/93.4	94.0/94.7	91.4/90.1
Complete (15,109/15,020)	82.9	85.4/84.9	90.5/83.2	90.1/83.0	89.4/82.7	99.0/83.1	86.0/91.2	86.2/85.6	85.2/89.9	84.5/92.6	82.1/82.5	81.7/82.2	82.7/81.4
Amino acids (length)													
nsp1*α* (180/180)	93.3	94.4/95.6	95.0/95.0	95.0/96.1	95.0/95.0	98.3/93.3	93.3/95.6	95.0/97.2	93.3/95.0	93.9/95.0	93.9/95.0	92.2/95.6	95.0/**96.7**
nsp1*β* (203/203)	72.9	82.8/79.8	89.7/73.4	89.7/73.4	86.7/75.9	99.0/72.9	78.8/82.3	88.2/74.4	78.8/82.3	74.4/85.7	76.4/76.8	78.8/74.4	77.8/82.8
nsp2 (1096/1065)	69.3	72.9/77.7	85.9/68.9	84.3/68.4	83.4/67.8	98.7/69.5	73.4/86.0	72.2/78.1	72.5/84.6	72.0/89.3	70.2/73.8	68.6/73.7	63.9/64.0
nsp2TF (919/888)	64.4	69.3/75.3	85.2/65.0	83.5/64.5	82.4/64.0	98.7/64.7	68.4/84.1	68.2/75.9	67.6/82.5	67.2/87.6	65.7/70.4	64.5/70.4	59.0/59.3
nsp2N (750/719)	57.7	64.3/72.2	83.5/58.9	81.3/58.4	80.3/58.0	98.4/58.1	62.8/81.9	63.2/72.6	61.7/80.0	61.2/85.8	60.7/66.8	59.3/66.8	53.5/53.9
nsp3 (230/230)	93.9	90.4/90.4	92.6/93.0	92.6/93.0	92.6/92.6	99.6/94.3	96.1/94.8	88.7/88.3	93.5/93.5	94.8/93.9	92.2/90.9	93.0/91.3	93.5/92.2
nsp4 (204/204)	91.7	90.2/91.2	90.7/91.7	90.7/91.7	90.7/91.7	99.5/92.2	93.1/96.1	90.2/93.1	92.2/95.1	92.6/98.0	88.2/89.7	90.2/91.7	**93.1**/93.6
nsp5 (170/170)	85.9	87.1/87.1	90.0/82.9	88.2/81.8	90.0/82.4	100/85.9	91.2/91.8	92.4/85.9	91.2/88.2	88.2/91.8	91.2/85.3	87.6/84.7	**94.1**/87.6
nsp6 (16/16)	100	100/100	93.8/93.8	93.8/93.8	87.5/87.5	100/100	100/100	100/100	100/100	100/100	100/100	100/100	**100**/100
nsp7*α* (149/149)	91.9	90.6/92.6	95.3/92.6	95.3/92.6	94.6/93.3	100/91.9	90.6/95.3	95.3/91.3	89.3/94.0	90.6/96.0	89.9/91.3	89.9/94.0	90.6/92.6
nsp7*β* (110/110)	74.5	84.5/80.0	90.9/77.3	91.8/77.3	90.9/78.2	98.2/74.5	77.3/88.2	86.4/73.6	78.2/88.2	76.4/90.0	73.6/70.9	70.9/70.9	81.8/77.3
nsp8 (45/45)	88.9	86.7/91.1	93.3/95.6	93.3/95.6	93.3/95.6	97.8/91.1	88.9/93.3	93.3/95.6	88.9/91.1	88.9/93.3	82.2/86.7	80.0/84.4	86.7/91.1
nsp9 (685/685)	94.3	96.5/95.6	97.1/95.9	96.9/95.8	96.1/95.0	99.3/94.6	95.9/97.4	97.1/95.3	95.9/97.2	95.2/97.5	94.3/94.2	94.7/94.2	95.3/95.6
nsp10 (441/441)	97.1	97.5/97.1	98.0/97.1	97.5/97.0	97.7/96.8	99.3/96.8	98.0/98.0	97.7/97.5	96.8/97.1	97.1/97.7	94.1/93.9	94.3/94.6	95.0/95.7
nsp11 (223/223)	91.9	92.8/92.4	94.2/94.6	93.3/93.7	93.7/94.2	98.7/91.9	94.2/95.5	94.2/92.8	94.2/93.3	93.3/96.9	92.8/91.9	92.8/92.4	94.6/93.7
nsp12 (153/153)	87.6	93.5/90.2	95.4/88.2	95.4/88.2	95.4/88.2	98.7/88.9	88.2/96.1	97.4/90.2	87.6/96.1	86.9/96.7	89.5/90.9	89.5/91.5	89.5/92.2
GP2 (256/256)	82.0	87.5/87.5	94.9/83.2	94.1/83.2	94.1/82.0	97.7/82.4	84.8/91.4	85.9/87.1	83.2/88.3	84.4/94.5	82.8/80.9	81.6/83.6	85.9/87.1
E (73/73)	89.0	90.4/93.2	97.3/89.0	97.3/89.0	97.3/89.0	100/89.0	90.4/93.2	90.4/93.2	91.8/89.0	87.7/95.9	89.0/86.3	89.0/89.0	87.7/87.7
GP3 (254/254)	77.6	79.5/81.1	93.3/81.5	92.9/81.1	89.0/78.0	97.6/79.5	82.3/89.4	82.7/88.2	82.3/89.4	78.0/90.2	79.5/78.7	82.7/79.1	81.9/79.9
GP4 (178/178)	92.7	87.1/86.5	97.2/93.8	97.2/94.4	92.1/89.9	97.8/93.3	95.5/93.8	93.3/92.7	95.5/93.8	96.1/94.9	88.2/87.6	87.6/86.5	86.5/87.1
GP5a (46/46)	91.3	93.5/91.3	97.8/93.5	97.8/93.5	97.8/93.5	100/91.3	93.5/93.5	91.3/95.7	93.5/93.5	89.1/95.7	95.7/91.3	84.8/84.8	87.0/84.8
GP5 (200/200)	88.5	90.5/87.0	97.0/88.0	96.0/88.0	95.0/87.5	99.5/88.0	91.5/89.5	90.5/88.0	91.5/89.5	90.0/92.0	90.5/86.5	90.5/85.0	84.5/81.5
M (174/174)	92.0	91.4/93.7	97.7/93.7	97.7/93.7	96.0/93.7	99.4/92.5	92.0/96.6	91.4/96.0	92.0/96.6	92.5/97.1	92.0/93.1	92.5/93.7	92.0/93.1
N (123/123)	92.7	90.2/91.9	96.7/93.5	96.7/93.5	96.7/91.9	100/92.7	97.6/95.1	94.3/93.5	97.6/95.1	95.9/96.7	90.2/91.9	91.9/91.1	91.9/93.5

*Note:* Bold values signify the nsps of VR-2332 with the highest amino acid identities compared to GNU-2353 and GNU-2377 in this study.

**Table 2:** Genetic recombination events of GNU-2353 and GNU-2377 detected by RDP4 software.

Strains	Methods	Breakpoints (nt)		Major parent (similarity)	Minor parent (similarity)	*p*-Value
		Beginning	Ending			
GNU-2353	RDP	5740	6862	NADC34 (91.1%)	RespPRRS MLV (93.9%)	2.365 × 10^−31^
	GENECOV^a^					3.710 × 10^−02^
	BootScan					2.668 × 10^−30^
	MaxChi					1.649 × 10^−12^
	Chimaera^a^					5.124 × 10^−06^
	SiScan					7.453 × 10^−22^
	3Seq					3.330 × 10^−16^
	LARD					1.153 × 10^−27^

GNU-2377	RDP	470	856	NADC30 (91.4%)	RespPRRS MLV (97.9%)	8.359 × 10^−17^
	GENECOV^a^					9.330 × 10^−06^
	BootScan					1.844 × 10^−16^
	MaxChi					1.586 × 10^−07^
	Chimaera					1.537 × 10^−08^
	SiScan					8.470 × 10^−12^
	3Seq					1.921 × 10^−12^
	LARD					2.639 × 10^−10^

^a^This method is inapplicable because *p*-value is higher than 1 × 10^−6^.

**Table 3:** Comparison of the whole-genome sequences of GNU-2353 and GNU-2377 from original P0 isolates and cell culture-passaged P1 and P5 strains.

Strains	Coding regions	Nucleotides				Amino acids			
		Positions	P0	P1	P5	Positions	P0	P1	P5
GNU-2353	nsp2	2872	C	T	T	958	L	F	F
		3070	G	A	A	1024	V	I	I
		3111	C	T	T	1037	–^a^	–	–
	nsp3	366	G	A	A	122	–	–	–
		495	C	T	T	165	–	–	–
	nsp4	63	C	T	T	21	–	–	–
		73	G	A	A	25	V	M	M
		183	A	G	G	61	–	–	–
		310	G	A	A	104	V	I	I
		317	A	G	G	106	K	R	R
		411	C	T	T	137	–	–	–
	nsp11	469	A	A	G	157	R	R	G
	GP2	251	C	C	T	84	T	T	M
		636	C	C	T	212	–	–	–
	GP3	13	C	C	T	5	R	R	C

GNU-2377	nsp2	1949	C	C	T	650	P	P	L
		2019	A	A	G	673	–	–	–
		2069	A	A	G	690	N	N	S
		2323	T	A	A	775	S	T	T
		2664	A	G	G	888	–	–	–
		2679	C	T	T	893	–	–	–
		2763	G	A	A	921	–	–	–
		2935	T	T	C	979	–	–	–
		3033	C	T	T	1011	–	–	–
		3057	C	T	T	1019	–	–	–
	nsp3	318	A	T	T	106	L	F	F
	nsp4	585	T	T	C	195	–	–	–
		588	G	G	A	196	–	–	–
		597	C	C	A	199	–	–	–

^a^No amino acid change found.

## Data Availability

The data that support the findings of this study are included within the article, and the obtained full-length genome sequences were deposited in the GenBank database.
